# Supplementary material for: Are carbon emissions trading and green financial instruments synergistic? -Comprehensive quantitative research based on content analysis
Source: PLoS One. 2024 Mar 7;19(3):e0298601. doi: 10.1371/journal.pone.0298601 (PMC10919601; doi:10.1371/journal.pone.0298601)
Supplement: S1 Data — (ZIP) [file pone.0298601.s001.zip › Data/地级市Z_F工作报告.zip!/║┌┴·╜¡/─╡╡ñ╜¡╩╨/└┤╫╘╣1⁄2╓┌║┼í╛┬φ┐╦╩2╛▌═°í┐_2006.docx]

各位代表：

　　现在，我受市人民政府委托，向大会作工作报告，请予审议，并请各位政协委员和列席人员提出意见。

　　一、2006年工作和过去四年发展的简要回顾

　　2006年，市政府在省委、省政府和市委正确领导下，在市人大、市政协监督支持下，认真践行“三个代表”重要思想，树立和落实科学发展观，带领全市人民拼搏进取，攻坚克难，实现了“十一五”发展的良好开局。

　　（一）经济平稳较快增长，综合实力持续增强。全市地区生产总值完成347.1亿元，同比增长12.8%。全社会固定资产投资101.2亿元，增长17.4%。规模以上工业增加值、销售收入和税金实现45.4亿元、160.5亿元和9.1亿元，分别增长12.1%、14.7%和5.3%。全口径财政收入32.6亿元，增长9.5%。城镇居民人均可支配收入和农民人均纯收入达到8035元和4686元，增长9.6%和14.7%，城乡居民储蓄存款余额394.1亿元，比年初增加25亿元。社会消费品零售总额134亿元，增长11.2%。

　　（二）改革重组和项目建设取得突破，工业经济增势明显。制药、炼油两户骨干企业当年重组、当年启动、当年见效，重组改制后的桦林等大企业和60%以上中小企业活力倍增，水泥集团重组迈出实质性步伐，牡纺等3户企业政策性破产获国家批准。23个市级重点项目有18个开工和续建，完成投资26.7亿元，其中7个工业项目完成投资7.7亿元，恒丰2万吨高档印刷型水松原纸、皓月20万头肉牛加工等项目投产，一批新项目进展顺利。全年开发新技术、新产品57项，高新技术产业产值达到62亿元。市商业银行挂牌运营。改组成立了牡丹江城市投资（集团）公司。整合后的市开发区建设步伐加快，12户投资5000万元以上的企业入区、5户开工建设。

　　（三）新农村建设实现良好开局，农业和农村发展形势喜人。投入新农村建设资金8.4亿元，新建农村水泥公路1113公里、乡镇客运站8个，新增安全饮用水人口8.6万人，涌现出海林农场、宁安小朱家村等新农村建设典型。种植业连续四年大丰收，粮食总产再创新高。四大主导产业收入占农业收入的“半壁江山”，主导产业支撑农村经济的格局初步形成，农村经济总收入实现153.8亿元，同比增长11.2%。设立了全省首家境外劳务输出服务中心，全市向域外累计转移农村劳动力14.7万人，落实国家惠农补贴资金3亿元，农民人均纯收入连续四年全省第一。绿化造林9.9万亩，治理水土流失35.1万亩，穆棱清河等三个“黑土区”、宁安响水灌区、镜泊湖水库除险加固工程列入国家农业基础设施建设投资计划。

　　（四）外经外贸继续攀升，旅游业呈现加速发展态势。全市外贸进出口总额实现62亿美元，同比增长30.2%，其中对俄贸易42.4亿美元，增长7.8%，分别占全省的48.2%和63.4%。对俄经贸产业带初具规模，发展势头强劲，绥——波贸易综合体一期工程落成运营，引进哈尔滨华晟国运物流公司，年木材进口量达到776万立方米，加工能力达到350万立方米。实施“走出去”战略，境外工业园区达到4个、企业12户。“俄罗斯年”活动丰富多彩，举办中俄边境地区经贸合作论坛等活动70多次，镜泊湖景区被联合国评为世界地质公园，首尔航班增至每周四班，韩国入境旅游者增长3倍，台湾旅游者翻了一番，全市旅游业总收入实现30亿元，同比增长16.4%，出入境旅游人数和外汇收入居全省首位。

　　（五）国内外区域合作不断深入，招商引资再结硕果。10个沪牡合作项目开工建设，到位资金6.7亿元，新签约3000吨多晶硅等22个项目，总投资48.7亿元，开展了教师培训和医疗技术协作，上海至牡丹江直飞航班复航，江海经贸发展有限公司启动运营，合作组建了龙裕城建开发公司，与浙江嘉兴市确定为友好市，与哈尔滨、大庆等地合作进一步深化。组织开展赴沪苏浙、香港、韩国、法国等招商活动，在韩国首尔设立全省第一家专业招商机构，“木博会”成为国家级常设展会，与韩港两地合作关系日益紧密，招商引资向产业集群、对俄进出口加工等领域集中，全年引进域外资金到位74.5亿元，同比增长19.3%，实际利用外资连续三年保持全省第二位，新签约亿元以上项目36个，其中16个开工、3个投产。

　　（六）保障水平进一步提高，和谐社会建设得以加强。率先在全省启动退休人员医疗保险，落实了1.7万名改制中小企业退休职工终身医疗保险，企业职工最低工资标准由305元提高到475元，城区低保金标准由120元提高到150元，改革了公务员工资制度和事业单位收入分配制度，全面实施了农村居民最低生活保障制度，半数以上农民参加新型农村合作医疗。新增就业岗位7.6万个，安置城镇失业人员6.4万人，为6850名符合条件的下岗失业人员发放社保补贴1344万元，城镇登记失业率控制在3.9%以内。投资近3000万元，整修了11条道路和人民公园、北山公园、火车站西广场。完成供热管网改造200余万平方米，大气环境优良天数达到313天，20件为民实事基本完成，爱民区社区服务中心被评为全国优秀社区服务中心。完善了安全防控和应对突发性事件综合体系，各类事故发生率平稳下降，安全度过森林防火和防汛期，加大了信访工作力度，“平安牡丹江”建设扎实推进，社会治安状况良好。

　　（七）以承办省运会为契机，各项社会事业协调发展。累计投入1.9亿元，新建北山体育中心，改造东四等7个体育场馆，体育设施投入超过建国以来各个时期的总和。省运会圆满举行，我市代表团荣获金牌总数第四名和体育道德风尚奖，城市形象和市民风貌全面展示。歌舞、戏剧等文化品牌影响扩大，群众体育、广场文化活动有声有色。整合了市区3所中学和4家卫生机构，师范学校晋升省幼专工作进入国家评审阶段，农村中小学D级危房改造全部完成，妇儿医院新楼投入使用。人口和计划生育、民族宗教、外事侨务、接待、统计、审计、民兵预备役、边防、人防、防震减灾、气象、档案、修志工作以及老区、老年人、残疾人事业取得了新成绩。

　　各位代表，市十三届人民政府任期结束。过去四年，是全市上下经受考验、奋力前行、不断求索、争取胜利的四年。四年间，全市地区生产总值年均增长9.7%，规模以上工业增加值、销售收入和利税年均增长11.9%、14.5%和11.6%，全社会固定资产投资年均增长15.2%，全口径财政收入年均增长13.3%，经济发展呈现逐步回升的良好态势。四年间，通过预算安排和争取上级财政资金，累计投入86亿元，推进了基础设施建设、江南开发、旧城改造和环境综合治理工程，城乡面貌进一步改观，道路交通条件有了根本改善，科技教育、医疗卫生和文化体育等事业取得了长足发展。四年间，社会保障体系不断完善，覆盖面不断扩大，保障能力不断增强，城镇居民人均可支配收入和农民人均纯收入分别增加2459元和1989元，人民生活质量进一步改善。四年间，市政府深入开展普法教育，始终坚持依法行政、阳光理政，主动接受市人大的依法监督、市政协的民主监督和社会各界的舆论监督，妥善办理人民代表议案建议286件、政协委员提案754件，各项工作比较好地体现了人民的意志。

　　各位代表，我们所取得的成绩来之不易，这是省委、省政府和市委正确领导的结果，凝聚了全市各方面的心血和汗水。在此，我代表市人民政府，向全市各族人民，向人大代表、政协委员、各民主党派、工商联、无党派人士、人民团体和社会各界人士，向中省直单位、驻牡部队和武警官兵及外来投资者，向所有关心支持牡丹江发展的各级领导和国内外朋友，表示崇高的敬意和衷心的感谢！

　　在肯定成绩的同时，我们也深刻地认识到，全市发展中还存在一些突出矛盾和问题：一是市域经济发展不平衡，中心城市特别是市直增长速度缓慢，实力不强。二是体制与机制障碍较多，投融资体制和开发建设体制改革滞后，诚信体系不健全。三是经济结构与布局调整任务依然较重，经济一体化格局尚未形成。四是人民生活质量和生活环境亟待进一步提高，部分群众生产生活困难，人才引进难、留住难的问题比较突出。五是经济发展环境还需进一步改善，政府部门的执行与落实能力、工作效能与管理服务水平需要进一步提高。对这些问题，我们一定高度重视，认真加以解决。

　　二、今后五年的目标任务和2007年主要工作

　　今后五年，是我市抢抓机遇、负重奋进、努力实现又好又快发展的关键时期，也是我市实施“十一五”规划、建设社会主义新农村和构建和谐社会的重要时期，人民群众期盼加快发展、渴望尽快富裕的呼声十分强烈。我们必须增强忧患意识、责任意识、赶超意识和争先意识，聚精会神搞建设，一心一意谋发展，抓住大事、解决难事、办好实事，全面开创各项工作的新局面。贯彻落实市十次党代会精神，市政府工作的总体思路是：高举邓小平理论和“三个代表”重要思想伟大旗帜，以科学发展观和构建和谐社会统领经济社会发展全局，以提高人民生活品质为出发点和落脚点，大力推进工业立市、商贸强市、旅游旺市、科教兴市战略，进一步扩大改革开放，加快社会主义新农村建设，统筹协调发展各项社会事业，保护生态环境，节约能源资源，围绕建设实力牡丹江、魅力牡丹江、和谐牡丹江，倾力打造现代加工业基地、东北亚区域商贸中心、全国著名旅游休闲度假胜地、黑龙江东南部区域中心和全省最适宜人居创业城市，不断完善城市功能，努力实现追赶型、跨越式发展。

　　按照经济总量、财政收入、人民生活水平“三个同步提升”和科学发展、和谐发展的总体要求，今后五年全市经济社会发展预期目标为：地区生产总值年均增长11%以上，突破600亿元，人均地区生产总值超过2万元；全口径财政收入年均增长10%左右，突破50亿元，其中，市本级和市直财政收入年均增长8%左右，分别达到20亿元和12亿元；城镇居民人均可支配收入年均增长8%，达到1.1万元；农民人均纯收入年均增长7%，达到6500元；社会消费品零售总额年均增长9%，达到220亿元；城镇登记失业率控制在5%以内，市区普及高中教育，社区卫生服务人口覆盖率达到98%以上；万元地区生产总值能耗年均下降4%；中心城区规划建设面积达到100平方公里，总人口达到100万人。在确保完成预期增长目标的基础上，我们要自加压力，做到好中求快、更好更快，力争五年内成为全省工业大市、财政强市和人均收入最高地区之一，达到全省地市上游水平，全国同等城市中游水平。

　　千里之行，始于足下。今年，我们要按照市委的统一部署，坚持一手抓当前、一手抓长远，既要敢闯敢干，更要讲求实效；既要立竿见影，更要增强后劲；既要注重发展，更要关注民生；既要发展速度，更要发展质量，务求在“全面提升一个龙头地位、努力突破五大发展瓶颈、着力抓好八项重点工作”上取得实效，确保全面完成或超额完成各项任务，全市地区生产总值增长12%，全社会固定资产投资增长25%，规模以上工业增加值、销售收入和税金增长13%、15%和11%，农村经济总收入增长6%以上，外贸进出口总额增长20%，全口径财政收入增长8%，其中市直财政收入增长6%，城镇居民人均可支配收入增长10%，农民人均纯收入增长7%以上。

　　全面提升一个龙头地位：

　　大力推进哈—牡—绥东对俄经贸产业带和陆海联运大通道建设。实施口岸牵动、哈—牡—绥东沿线联动，加快打造省内新的经济增长极。明晰产业带发展战略。近期着力推进“牡海宁”、“绥（东）穆（林）”两极互动，把木业作为产业带发展的切入点、支撑点和增长点，发挥优势，突出特色，迅速形成规模效益。中远期实现“双向延伸”，向内建设与哈大齐工业走廊比翼齐飞的全省开放带、经济带、旅游带、文明带；向外连接俄罗斯乌苏里斯克、海参崴等城市，发展跨国经贸科技合作区。整体推进产业带建设。坚持整合规划、共享品牌、协调政策、错位发展，开发建设产业带沿线“一区”、“六园”。依托木材、纸浆、石油等进口资源，突出抓好以市开发区、绥—绥工业园、东宁浙江工业园和海林木业城等园区为重点的木业产业平台，加快建设以绥芬河、东宁为前沿，绥阳、下城子、市区和海林为腹地的进口加工基地，壮大牡丹江、绥芬河、东宁三个省级对俄出口加工基地，优先发展俄罗斯需求较旺的轻纺、机电、食品、建材及装饰材料等产业。今年，产业带新开工5000万元以上项目29个，其中市开发区开工10个。提升大通道功能。立足产业带、服务哈大齐，确保“借港出海”、出境不出口陆海联运大通道正式运营，开通黑龙江省新的出海口。新建绥芬河铁路口岸客运候车大厅，绥芬河国际客运站和铁路电子口岸投入使用，启用绥芬河铁路液体换装线，建设绥芬河至下城子铁路复线，争取东宁口岸界河桥、东—波互贸区外交换文，推进东—乌国际铁路建设实质性运作，实施牡丹江机场改造工程，着手制定新机场建设规划。大力发展物流园区和专业物流中心，抓好华晟国运物流、绥芬河国际物流、海林华运物流和下城子物流中转集散中心建设。

　　努力突破五大发展瓶颈：

　　（一）在盘活存量、导入增量、扩张总量上实现突破。围绕当期增效益、长远添后劲，全力保持固定资产投资合理较快增长。加快企业改制重组。确保水泥集团重组成功，完成牡纺等3户企业政策性破产，继续争取桦林集团等企业政策性破产获国家批准，活化绿津实业、牡康等企业闲置资产，启动厂办大集体企业改制试点，推动改制企业解决遗留问题、不断靠大联强。促进项目批量落地。确定投资规模5000万元以上的市级重点项目54个，总投资466亿元，其中工业项目23个。今年计划投资41亿元，确保4万吨醋酸乙烯改扩建等13个项目竣工投产，续建460万套子午胎等16个项目，“双16万吨”烧碱和树脂等10个项目开工建设，3000吨多晶硅等7个项目争取开工，加快3万吨海绵钛等8个项目前期运作。全力抓好“上争外引”。做实项目谋划和储备工作，积极落实项目配套资金和建设条件，为向上争取和招商引资打好基础。把引进重大项目和战略投资者作为主攻方向，强化政府搭台、企业主体的双重作用，巩固韩国、香港和长三角、珠三角招商市场，积极拓展欧美等招商市场，开展多种形式的务实招商，抓好签约项目跟踪问效，扩大招商成果。全年引进亿元以上项目22个，域外资金到位85亿元，实际利用外资7400万美元，同比分别增长15%和18%。

　　（二）在资源整合、区域联动、整体发展上实现突破。强化大牡丹江观念，坚持行政手段与市场手段相结合，加快区域经济一体化进程。加大重大资源整合开发力度。成立全市重大资源开发项目专题推进组，对市域内水能、风能、矿产、林木等资源整体规划、统一运作、合理开发，降低运作成本，提高资源开发利用率和资源性项目招商落地率。创新利益联接机制。以效益为纽带，制定区域内资源共享、政策共享、平台共享、利益共享的措施办法，打破区域、条块界限，促进市县区之间、地方与农垦和森工等中省直单位之间的合作，构筑区域经济整体优势。按照重心下移、责权利相一致原则，明确事权划分，建立“两级政府、三级管理、四级网络”的城市管理体制。放大园区集聚效应。坚持项目园区化、园区专业化，制定园区产业发展指导目录，进一步规范园区发展，优化园区布局，细化明确各园区功能定位，构建以市开发区为龙头的国家、省、市三级园区体系，积极发展以区中园、园中园为主要形式的“飞地经济”。迅速壮大县区经济。深入贯彻落实省委《关于进一步加快县域经济发展的决定》，支持绥芬河、东宁口岸县（市）率先发展，鼓励绥芬河加快“北方深圳”建设，扶持内陆县（市）发挥比较优势，发展特色产业，围绕全国“百强”、全省“十强”目标，升位争先。各城区也要因地制宜，走主导产业立区、招商引资兴区的路子，扩大城区经济规模，增强发展活力与实力。

　　（三）在完善投融资和城市开发体制、提升资本运作水平上实现突破。以创新体制机制为前提，以市场化运作为核心，畅通融资渠道，有效破解资金瓶颈。构筑稳固的投融资平台。深化投融资管理体制改革，加大资源、资产、资本“三资”注入力度，做大投融资平台。严格履行借、用、管、还责任，强化对项目运作、资本金及投融资的调控，理顺开发建设体制，加快城市开发建设进程，提高建设资金使用效益。建立多元化融资机制。加强信用建设，营造良好的金融生态环境，巩固与开发银行和各商业银行的合作关系。深化农村信用社改革，扶持城市商业银行发展，壮大地方金融机构实力。积极引进外资银行和国内股份制银行分支机构以及保险、信托、证券、基金等其他金融机构，发行企业债券，活跃民间资本，扶持有条件的企业上市融资。全方位搞好城市经营。利用城投集团公司平台，坚持储备统一渠道、开发统一运作、收益统一监管的“三位一体”模式，对城市基础设施和公益类、资源性项目以及政府承贷项目进行整体包装和市场化运作，提高成功率。

　　（四）在培植壮大财源、建立健全公共财政体制上实现突破。完善“生财、聚财、理财、用财”措施，逐步建立以民生为本、有利于各项事业协调发展的公共服务均等化财政分配体制，为构建和谐社会奠定坚实的财力基础。大力培植各类财源。加速骨干企业改制重组步伐，清理规范财税政策，统一政策标准，公平发展环境，整合各类财源建设资金集中打捆使用，做大存量财源。通过招商引资、项目建设，发展“总部经济”、“结算经济”和“参股经济”，培植新生财源。积极争取国家和省政策性资金及转移支付资金，扩充政策性和体制性财源。完善市、县（市）、区财源项目税收分享办法，扶持共享财源。坚持依法从严治税，强化重点税源监控，建立市直税收监管机构和全市税源管理信息共享平台，防止税源流失，稳固既得财源。加强非税收入管理，逐步将国有资本收益、国有资源有偿使用和特许经营等收入纳入管理范围，拓展非税财源。深化财政管理体制改革。巩固部门预算、综合预算、国库集中收付、政府采购制度等改革成果，全面推进政府收支分类改革，实施国有资本经营预算管理制度，推行机关后勤服务社会化、供热货币化改革，探索公车改革，认真做好统一内外资企业所得税准备工作。按照省里要求，年底前完成事业单位综合配套改革。严格财政资金监督管理。完善监督机制，对财政资金进行全程监督，发挥财政投资评审作用，提高投资评审质量，确保财政资金运行规范安全。做好项目绩效综合评价工作，提高财政投资和支出效益。出台债务管理办法，规范政府举债、使用、偿还和担保行为，完善政府债务风险预警和准备金制度，保证到期债务及时偿还。提高公共财政保障能力。优化财政支出结构，加大新农村建设、就业再就业和社会保障、社会事业发展和生态环境保护投入力度。认真落实党政机关、事业单位、离退休人员工资改革和套改、发放地区津贴等政策措施。完善住房公积金补贴资金发放制度，对符合条件的职工及时发放住房补贴。推行廉租住房保障制度，进一步改善城镇低收入困难群众住房条件。

　　（五）在经济结构和布局调整、增强发展活力上实现突破。高度重视经济社会发展规划，坚持工业化、产业化与城市化互动，坚持用规划推动建设和管理，提高经济结构层次，优化生产力空间布局，培育壮大新的经济增长点。合理确定产业布局。依据城市功能定位，打破行政区域界限，加快建设以进出口加工基地和商贸物流中心为主的中部板块，以精品旅游为主的西部板块，以森林及矿产资源加工为主的北部板块，以生态及现代农业加工为主的南部板块。遵循两大城市组团布局，以实现“双百”为目标，拓展城市发展空间，加快户籍制度改革，优先做大中心城市，提高人口承载和产业集聚能力；加快打造“绥东绥”城镇化组团，配套发展卫星城，协调发展小城镇，有序发展新农村，形成外向型、加工型、旅游型、商贸型特色小城镇体系。调整优化工业结构。改造传统优势产业，整合提升大庆路工业园区，再造牡丹江工业新优势。围绕做大森林工业、汽车配套、石油化工、新型材料、医药、能源六大产业，推进产业资源整合，延伸产业链。巩固扩大恒丰、大宇等纸业企业规模和市场份额；扶持桦林佳通、富通等汽车配套企业，提高整体配套能力和产业层次；通过重组、改造和上新项目等途径，形成以东北化工、东北高新等企业为支撑的化工产业；以高科生化为龙头，开发整合“北药”资源，增强药业企业整体竞争力；加快发展木业产业，提高木材深加工水平；以多晶硅项目为牵动，促进圣戈班、顺达电石等企业提档升级。围绕食品工业、农业资源深加工、进出口加工、高新技术产业、绿色能源和循环经济，大力发展新型工业。壮大旅游和会展经济。以“对俄大通道、生态牡丹江”为主题，精心打造镜泊湖、林海雪原和百年口岸三大品牌，扩大镜泊湖世界地质公园影响，争取镜泊湖成为国家5A级景区，创办“镜泊湖之夏”旅游节，丰富冬季冰雪特色旅游项目，大力兴办乡村旅游。强化旅游市场营销，拓展旅游客源市场，规范提高旅游业服务水平，全年旅游人数和旅游业总收入实现两位数增长。推进“十大旅游精品工程”建设，深度开发特色旅游商品。依托产业带、立足牡丹江、辐射黑龙江、面向东北亚，精心承办第三届中国（牡丹江）木博会，办好首届牡丹江雪堡经贸旅游洽谈会、中国（牡丹江）农业产业经济高峰论坛暨特色农产品博览会、中国（东宁）黑木耳节和绥芬河元宵灯节等展会活动，规划筹建牡丹江国际会展中心。优化提升城市商贸商务功能。坚持功能错位、突出特色，调整商业网点和布局，提升发展中心城区零售商业，规范发展社区商业和地区商业副中心，加快发展交通枢纽型商业，创新发展批发贸易业，培育发展生产型服务业。规划建设东安中央商务区、西安民族商业区、阳明汽贸服务区、爱民大学城服务区和江南行政服务区。积极发展金融服务业、现代房地产业、第三方物流、行业协会和中介组织，培育休闲、健身、文化等新型消费服务业，不断改善城乡消费环境，促进居民消费升级。扶持非公有制经济发展。放大工业发展专项资金调节作用，建立中小企业互助基金，积极争取国家和省科技专项资金、贷款贴息，发展“两台一会”，增强中小企业融资能力。实施“中小企业成长工程”，辟建江南中小企业创业园，扶持劳动和技术密集型等民营企业发展，形成专精特新、机制灵活的中小企业群体。全市非公有制企业实缴税金增长12%，市区20户“小巨人”企业销售收入增长20%，新增30户以上规模工业企业。

　　着力抓好八项重点工作：

　　（一）以对俄、对韩合作“双升级”为牵动，全方位扩大对外开放合作。充分利用国际国内两个市场、两种资源，在推进对俄经贸科技合作的同时，加速对韩合作战略升级，扩大与其他周边国家和国内城市跨区域合作，增强市域经济发展的外部动力。推进对内对外合作。对内以上海和省内城市为重点，对外以俄罗斯、韩国为重点，加快建设跨区域合作的前导区、示范区。大力度推进沪牡合作，全方位对接合作项目，进一步创新合作模式，拓展合作领域。加强与省城及省内各地市合作，积极参与黑龙江东部煤电化基地建设，建立与周边城市的战略合作伙伴关系。完善对俄合作规划，着手制定对韩合作规划，以进出口加工为导向，按照“中中外”、“中外中”模式，办好境外工业园，辟建绥芬河远东工业园和乌苏里斯克康吉经贸合作区，联合境内外企业和引导本地企业与俄方开展资源能源开发及种养业、旅游、房地产开发、建筑工程承包、金融、高新技术等领域的合作。办好中俄信息产业园，探索引进韩国企业建设韩国工业园。发展对外贸易。以专业化、现代化为方向，引导绥——波、东——波互贸区错位经营，完善绥芬河青云、迎泽和市区朝鲜民族商场等专业市场功能，促进哈巴罗夫斯克亚洲国际商贸城投入运营。转变贸易增长方式，提高地产品、农产品、机电产品和高科技产品出口规模，扩大能源、原材料等进口规模，积极培育省级出口品牌。巩固传统市场，拓展欧美等发达国家地区市场，开拓南美和非洲市场，构建贸易伙伴多元化格局。扩大对外交流。抓住“俄罗斯·中国年”和“2007中韩交流年”的有利契机，在俄罗斯远东地区和韩国首尔、坡州等城市全面宣传展示牡丹江，推动对俄对韩经贸、科教、文体等各领域交流合作，掀起“牡丹江热”。落实与俄罗斯乌苏里斯克、韩国坡州等友好城市定期互访机制，促进友好城市向合作城市发展。

　　（二）大力发展现代农业，奠定新农村建设的物质基础。以产业化经营牵动现代农业发展，推动农业不断增效、农村加快发展、农民持续增收。培育现代农业产业体系。优化农业产业结构和生产布局，做大做强四大主导产业和特色养殖、园艺等产业，引导“一村一品”向优势产业集聚，促进农业向产业化、规模化、区域化方向发展，四大主导产业收入占农业总收入的比重达到55%以上。发展农产品精深加工，壮大皓月、鑫鹏、三通、草原兴发等加工型龙头企业，再培育一批省和国家知名农产品品牌。抓好10万头无公害肉牛、10亿袋（块）绿色有机食用菌、10万亩对俄绿色果菜、10万头无公害生猪四大产业基地建设，创建穆棱肉牛、东宁黑木耳、宁安绿色水稻三个国家级农业标准化示范区。提高现代农业综合生产能力。实施科技入户工程，全面推进农业科研机构、大专院校、科技推广部门、农垦与市县乡开展农业科技共建，创新农技推广机制，大力推广工厂化农业生产、测土配方施肥、新型能源等新技术。推进土地适度规模经营，土地流转达到100万亩。提高现代农业装备水平，田间作业综合机械化程度达到78.5%。加强农业基础设施建设，推进国家级响水大型灌区、镜泊湖水库除险加固、牡丹江左右岸防洪工程和“黑土区”水土流失综合防治等工程，治理水土流失面积26万亩。完善现代农业服务体系。健全以批发市场为中心、集贸市场和零售经营为基础的农产品流通网络，发展壮大绥阳黑木耳、双合果菜、东宁哈达等一批年交易额超亿元的专业市场，建设黑龙江省对俄农业科技合作园区，加快农产品进军国际市场步伐，地产农产品出口额达到3.5亿元。引导扶持农民和龙头企业兴办各类专业协会、合作社、股份公司等合作组织，提高农民组织化程度。实施农村实用人才培养工程，积极开展装备之冬、阳光培训、百万中专生培训等工作，培训农村实用人才30万人次，新转移农村劳动力1.8万人。在发展生产的同时，继续改善农村生产生活条件，抓好新农村试点，推进康居乡镇、康居新村和少数民族乡村建设。

　　（三）实施开发与改造互动，提升城市建设管理水平。坚持国家环保模范城、卫生城和山水园林城“三城同创”，改造老城、开发江南、集聚产业、连片发展，增强城市承载能力。加强城市基础设施建设。江南新城区兴隆组团基础设施工程实现“水通路畅成环闭合”，市开发区北区今年全部完成规划范围内基础设施建设，市民广场、市一中新校区等项目投入使用，推进东四跨江桥二期工程建设，力争开工建设远程物流五星级宾馆等一批新项目。建设城市规划展示馆、博物馆、革命烈士馆和青少年宫等公共设施，加快桥北等区域棚户区改造和整体开发。整修城区道路，打造光华街样板街路，抓好城区照明续建工程，力争实现主城区大街小巷全部有路灯，加强基础设施亮化、楼体亮化、牌匾广告亮化，营造城市夜色景观。健全城乡交通网络。围绕增强区域性交通枢纽功能、构建现代城市交通网络，开工建设牡绥高速公路、鹤大公路牡丹江至宁安段、鸡图公路老黑山至省界段、鸡讷公路林口至大罗密段，确保年内实现绥满公路虎峰岭至海林界段、鹤大公路宁安至杏山段及杏山至省界段竣工通车。综合整治城市环境。抓好“三山、三花、两树”绿化工程，打造沿江、沿山景观带，开展市花、市树评选活动。实施工业污染、低空面源污染和大气污染治理攻坚战，推进小锅炉并网，大力推广民用型煤，大气环境优良天数保持在300天以上。启动市污水处理厂，建设海林和宁安污水处理厂，争取林海水库供水工程开工，确保居民饮水安全。认真落实城市属地化管理权限，完善城管执法目标化管理和公安、城管联动机制，集中开展以交通秩序、户外广告、占道经营、违章建筑为重点的市容市貌综合整治活动。完善物业管理企业、业主委员会、居民委员会和公安派出所“四位一体”管理体制，提高物业管理水平。改造城市生活垃圾收运系统，推行分类收集管理，做好日常保洁工作，美化城市环境。

　　(四)加快创新型城市建设，推动经济社会可持续发展。坚持把增强自主创新能力作为转变经济增长方式的中心环节来抓，靠创新构筑竞争优势。发挥人才保障作用。完善人才工作政策措施，调动现有人才积极性，加大“招商引智”力度，培养引进一批科技拔尖人才和领军人才。充分发挥“外脑”作用，借助外地人才的力量，帮助我市提高创新能力。深入实施职工素质工程，广泛开展科普活动，不断提高劳动者技能。鼓励各领域和行业开展创新活动，营造尊重劳动、尊重知识、尊重人才、尊重创造的社会氛围。强化科技支撑能力。围绕电子信息、生物技术与医药、先进制造业与化工、新能源与节能环保四大领域，重点抓好特种材料和石油钻采装备两个国家“火炬计划”产业基地建设，培育恒丰、金刚钻碳化硼等一批高新技术企业集团，支持企业与科研院所开展技术联盟，不断研发新技术、新产品，大力培育企业品牌，加强知识产权保护。全年实施省级以上重点科技攻关项目30项、高新技术产业化项目20项，新增高新技术产品10项、省级以上高新技术企业5家，高新技术产业产值突破70亿元。加快发展循环经济。全面组织实施循环经济园区试点方案，围绕煤化工、造纸、热电联产等产业谋划和申报项目，积极争取国家和省支持，确保一批项目开工建设。充分利用试点政策，完成5个省级和30个市级试点企业废水治理和综合利用项目建设。按照“减量化、再利用、资源化”原则，制定全市循环经济发展规划，推进循环经济向宽领域、深层次发展。

　　（五）统筹发展社会事业，增强社会发展活力。以满足人民群众公共服务需求为重点，优化社会资源，促进社会进步，为和谐社会建设提供有力支撑。优先发展教育事业。全面落实农村义务教育经费保障机制，完善教师援助制度，建好农村寄宿制学校，不断提升农村教育资源配置水平，推动城乡教育均衡发展。以强化城市服务功能、建设区域性教育中心为目标，推行“四个一流”示范工程，组建幼儿教育中心，进一步改善各类学校办学条件，巩固提高省级示范高中办学水平，推进市朝中和穆棱一中争创省级示范高中，鼓励社会力量办学，积极引进国内各层次名校创办分校，做大做强牡丹江大学，支持在牡高校发展，规划建设大学城，创办教育产业园。大力发展职业教育，支持市高级技工学校申办技师学院，加快建设一批职业教育实训基地，满足社会就业创业需求。强化公共卫生保障。加强农村卫生院建设，全面推行农村新型合作医疗制度。调整区域卫生机构设置，扩大社区卫生服务覆盖面，降低群众医药消费支出，为群众提供良好的基本医疗服务。深化与国内知名医院交流合作，发展特色专科医院，培育名医名院。完善公共卫生体系，提高公民卫生保健意识，有效预防各类传染病。繁荣各项社会事业。加快发展文化事业和文化产业，活跃广场文化、社区文化等城乡群众性文化活动，壮大戏剧、歌舞等演出团体，创作文艺精品，开展历史遗迹保护和非物质文化遗产普查，完善传媒集团文化改革试点，抓好广播电视数字化工程建设。推进北山体育中心等场馆市场化经营，为开展全民健身活动创造条件。广泛开展拥军优属、拥政爱民活动，争创全国双拥模范城“五连冠”，抓好第二次农业普查，做好人口和计划生育、民族宗教、外事侨务、接待、审计、民兵预备役、边防、人防、防震减灾、气象、档案、修志等工作和老区、老年人、残疾人等事业。

　　（六）注重解决民生问题，保持社会和谐稳定。想群众之所想，急群众之所急，切实维护好、实现好、发展好人民群众的切身利益。扩大就业再就业。建立健全劳动力市场服务体系、劳动保障工作平台体系、再就业技能培训体系和下岗失业人员保障机制，重点解决好“4050”人员和“零就业”家庭就业问题，新增城镇就业6万人，实现再就业5万人，做好大学毕业生就业和复员转业军人安置工作，加强劳动监管和劳动争议调处，构建和谐的劳动关系。增强社会保障能力。大力推进以社会保险和城市低保制度为基础、专项救助相衔接、临时救助为补充、优惠政策和社会互助相配套的社会保障及救助体系建设，发展慈善事业，加强福利设施建设，加大对社会困难群体帮扶力度，引导社会力量兴办扶老、救孤、助残、济困等社会福利机构。落实《妇女儿童发展规划纲要》，保障妇女儿童合法权益。完善信访体制机制。组建市信访工作委员会、县（市）区群众工作局和乡镇（社区）信访调处中心，搭建三级信访工作平台。成立市联合接待中心和综合受理中心，设立协调督查办公室。构建人民调解、司法调解和行政调解“三位一体”工作体系，健全完善社会矛盾和纠纷调解机制。开展“信访攻坚行动”，集中解决好群众反映强烈的信访问题，努力做到解决一个问题，形成一项制度，化解一批矛盾。推进“平安牡丹江”建设。落实安全生产责任制和一票否决制，健全安全生产监管网络，强化生产企业、道路交通、建筑工地及各类危险源、事故源的监管，严防关停小煤矿反弹，坚决防止重特大安全事故发生。加强应急设施建设，完善突发事件应急体系，提高应急处置和公共安全保障能力。健全社会治安网格化管理体系，建立刚性务实的警力下沉机制，加强对重点部位和重点人群的管理，严厉打击各种犯罪行为，增强市民的安全感。举办首届“社区节”等活动，促进邻里和睦、社区和谐，打牢稳定和谐基础。

　　(七)优化经济发展环境，打造投资创业热土。从影响环境的关键环节和突出问题入手，标本兼治，重在治本，为全民创业、招商引资、加快发展扫清障碍、创造条件。提高行政审批效率。对外来投资项目实行领办代办、限时办结、超时默认制度，重大项目实施规划、立项、土地、环保“四审”联动和市级领导代办制，加快项目落地。加强行政审批服务中心建设，制定更加有力的约束措施，彻底解决体外循环和效率不高问题。规范收费检查行为。发挥市优化办的作用，严格审批针对企业进行的检查活动，建立集中定期收费制度，未经市优化办许可一律不得到企业检查收费。开通“市长环境热线”，从严惩处乱收费、乱罚款、乱摊派等行为，全力为企业营造“保护伞”。深化行政审批制度改革，最大限度地减少和规范审批事项，实行“阳光收费”。强化跟踪服务。对引进企业和落地项目，实行“保姆式”、“全天候”服务，多为企业解忧，不给企业添乱，及时解决项目建设和企业生产经营中遇到的各种困难。完善“双评”措施，企业、部门、群众和中介机构共同参与行风政风监督测评，促进市直及中省直部门积极主动地为企业服务。优化信用环境。深入开展“诚信政府、诚信企业、诚信社会、诚信市民”创建活动，加快建设社会信用体系，建立信用评价、守信激励和失信惩戒机制，着力打造诚信牡丹江。营造亲商创业氛围。视投资者为“上帝”，视纳税人为“衣食父母”，建立政府与本地企业和外商联系制度，定期开展联谊活动，邀请企业经营者参加政府的重要会议，及时听取企业对政府的意见和建议。把创业作为富民的根本之策，完善扶持政策，放宽前置条件，鼓励群众兴家业、能人办企业、干部创事业。

　　（八）全面加强政府自身建设，为加快发展提供有力保障。致力于建设责任政府、服务政府、法治政府，以求真务实、真抓实干的工作作风取信于民，以立言立行、雷厉风行的实际行动取信于民，以勤政为民、廉洁自律的政德政风取信于民，以经济实力增强、人民生活富裕的发展业绩取信于民。牢固树立以人为本的施政理念。坚持发展依靠人民，最大限度地调动广大群众投身发展的积极性、主动性，凝聚全市各方面力量，推动牡丹江跨越式发展。坚持发展为了人民，做任何决策都要以群众满意不满意、高兴不高兴、答应不答应为前提，干任何事情都要以民为重、以民为上、以民为先；坚持发展惠及人民，把雪中送炭与锦上添花有机结合起来，每年集中办好一批利民惠民实事，下大力气解决好群众最关心、最直接、最现实的利益问题，让全市人民充分享受改革发展的各项成果。坚持依法行政、开展绩效评估。把依法用权、依法办事贯穿于政府工作的各个方面、各个环节，全面落实行政执法责任制和行政执法过错责任追究制，做到严格执法、公正执法、文明执法、高效执法。自觉接受人大依法监督、政协民主监督和社会各界的舆论监督，认真办理人大议案、建议和政协提案，全面推行政务公开，确保政府各项决策民主、科学、规范。抓好效能监察和绩效评估，认真监督检查政府机关和公务员履行职责的情况，严肃查处推诿扯皮、不负责、不作为的行为，从严治政，不断增强政府的执行力、落实力、公信力。提升社会管理和公共服务水平。按照转变职能、权责一致、强化服务、改进管理、提高效能的要求，加快推进政企分开、政资分开、政事分开、政府与中介组织分开，把政府工作的重心从单纯抓经济发展、单纯追求经济增长速度真正转移到经济调节、市场监管、社会管理和公共服务上来。围绕增强社会文明度、经济富裕度、环境优美度、资源承载度、生活便宜度和公共安全度，创新管理和服务模式，拓宽服务领域，改进服务质量，不断提高人民群众对政府的满意度。加强公务员队伍建设。深入开展学习型机关、学习型干部队伍创建活动，提高公务员业务素质，增强各级政府领导干部驾驭工作全局、领导科学发展、推动改革创新、处理复杂问题的能力和水平。强化工作目标管理考核，激励先进、鞭策后进。建立完善廉政建设责任制、行政监察、财政监督和审计监督等制度，坚决遏制各种不正之风，严肃查处违反行政纪律和侵害群众利益的案件，坚决做到用制度管权、靠制度管人、按制度办事。

　　各位代表！加快发展、造福人民，是新一届政府必须认真履行的神圣职责。让我们在市委的坚强领导下，凝聚275万牡丹江各族人民的智慧和力量，解放思想，创新创业，锐意进取，奋发图强，竭尽全力地把我们的宏伟蓝图变为现实，共同拥抱牡丹江全面振兴的美好春天！
